# Influence of Photoplethysmogram Signal Quality on Pulse Arrival Time during Polysomnography

**DOI:** 10.3390/s23042220

**Published:** 2023-02-16

**Authors:** Mantas Rinkevičius, Peter H. Charlton, Raquel Bailón, Vaidotas Marozas

**Affiliations:** 1 Biomedical Engineering Institute, Kaunas University of Technology, K. Baršausko Str. 59, LT-51423 Kaunas, Lithuania; 2 Department of Public Health and Primary Care, University of Cambridge, Cambridge CB2 1TN, UK; 3 Research Centre for Biomedical Engineering, University of London, London WC1E 7HU, UK; 4 Biomedical Signal Interpretation and Computational Simulation (BSICoS) Group, Aragon Institute of Engineering Research (I3A), IIS Aragon, University of Zaragoza, 50009 Zaragoza, Spain; 5 Biomedical Research Networking Center (CIBER), 50018 Zaragoza, Spain; 6 Faculty of Electrical and Electronics Engineering, Kaunas University of Technology, Studentų Str. 50, LT-51368 Kaunas, Lithuania

**Keywords:** electrocardiogram, R wave, T wave, photoplethysmogram, pulse onset, PAT, SpO2, polysomnography, obstructive sleep apnea, hypopnea, irregular heart rhythm

## Abstract

Intervals of low-quality photoplethysmogram (PPG) signals might lead to significant inaccuracies in estimation of pulse arrival time (PAT) during polysomnography (PSG) studies. While PSG is considered to be a “gold standard” test for diagnosing obstructive sleep apnea (OSA), it also enables tracking apnea-related nocturnal blood pressure fluctuations correlated with PAT. Since the electrocardiogram (ECG) is recorded synchronously with the PPG during PSG, it makes sense to use the ECG signal for PPG signal-quality assessment. (1) Objective: to develop a PPG signal-quality assessment algorithm for robust PAT estimation, and investigate the influence of signal quality on PAT during various sleep stages and events such as OSA. (2) Approach: the proposed algorithm uses R and T waves from the ECG to determine approximate locations of PPG pulse onsets. The MESA database of 2055 PSG recordings was used for this study. (3) Results: the proportions of high-quality PPG were significantly lower in apnea-related oxygen desaturation (matched-pairs $r_{c}$ = 0.88 and $r_{c}$ = 0.97, compared to OSA and hypopnea, respectively, when *p* < 0.001) and arousal ($r_{c}$ = 0.93 and $r_{c}$ = 0.98, when *p* < 0.001) than in apnea events. The significantly large effect size of interquartile ranges of PAT distributions was between low- and high-quality PPG (*p* < 0.001, $r_{c}$ = 0.98), and regular and irregular pulse waves (*p* < 0.001, $r_{c}$ = 0.74), whereas a lower quality of the PPG signal was found to be associated with a higher interquartile range of PAT across all subjects. Suggested PPG signal quality-based PAT evaluation reduced deviations (e.g., $r_{c}$ = 0.97, $r_{c}$ = 0.97, $r_{c}$ = 0.99 in hypopnea, oxygen desaturation, and arousal stages, respectively, when *p* < 0.001) and allowed obtaining statistically larger differences between different sleep stages and events. (4) Significance: the implemented algorithm has the potential to increase the robustness of PAT estimation in PSG studies related to nocturnal blood pressure monitoring.

## 1. Introduction

Photoplethysmography (PPG) is a non-invasive technology that enables tracking changes of human blood volume in peripheral blood vessels in order to assess hemodynamic activity. In clinical practice, PPG is commonly used in pulse oximeters to estimate arterial blood oxygen saturation (SpO2) [1,2,3,4]. Variations in SpO2 are often observed during full night polysomnography (PSG) studies [5,6,7,8]. The PSG test is considered the “gold standard” for diagnosing obstructive sleep apnea (OSA) and other sleep-related breathing disorders [9]. During PSG, many different biosignals are recorded to extract clinically relevant information on sleep apnea. For instance, fluctuations in blood pressure can be tracked [10,11,12,13], as these are known to be associated with OSA [12,14,15,16,17]. Furthermore, nocturnal blood pressure can be an independent predictor of cardiovascular events [18] providing useful information on cardiac activity. For long-term monitoring purposes, a commonly applied method to indirectly estimate blood pressure response involves the extraction of pulse arrival time (PAT) [11,12,13,19]. PAT is defined as the time delay from the R wave peak of the electrocardiogram (ECG) to the fiducial point of the PPG pulse waveform [13]. One of the technological solutions that uses the combined analysis of ECG and PPG signals for cuffless blood pressure assessment is the SOMNOtouch NIBP device [20,21]. However, in study [22] it demonstrated poor agreement with a validated oscillometric device during 24-h ambulatory blood pressure monitoring. Based on this, we assume that these large deviations in PAT can appear in part due to the poor quality of the PPG signal during different sleep stages and disturbances such as OSA. During pathological sleep, PPG signals might be sensitive to motion artifacts, micro-arousals, OSA-related body motions, or altered hemodynamics due to cardiac arrhythmia. Consequently, it is important to exclude low-quality PPG segments from such analyses before extracting clinically relevant information to ensure accurate diagnoses.

Many algorithms have been suggested to assess the quality of the PPG pulse waveform [23,24,25,26,27,28,29]. For instance, Elgendi [30] investigated eight methods to evaluate the quality of the pulse waveform and found that the skewness of the signal is an optimal index for this purpose. However, the estimation of higher-order statistics such as the skewness requires a longer time window than other metrics. Thus, it is less suitable for the analysis of short signals, e.g., a single-pulse waveform or during sleep events such as OSA. In addition, the method should be fast as PSG signals are always long. Another frequently applied method calculates a correlation coefficient between PPG pulse waves and a template pulse wave [28,29]. In this case, the estimation of correlation between pulse waveforms can require a resampling procedure for each PPG pulse [31], which is not very computationally efficient.

Other PPG signal quality indexes take into account limits on physiological viability, such as mean heart rate [28], maximum pulse-to-pulse interval [28], the ratio between maximum and minimum pulse-to-pulse intervals [28], instantaneous pulse-rate tracking [32], or pulse-rise time duration [27]. However, these physiological features can vary widely during cardiac arrhythmia, often seen in PSG signals [33,34,35]. Utilizing this type of technique may increase the probability that irregular rhythms (such as atrial fibrillation) could result in PPG pulse waves being incorrectly classified as artifactual.

OSA has been found to be associated with the development of cardiovascular disease and impaired cardiac function [33,34,35,36,37,38]. Therefore, it may be expected that periods of irregular rhythm will occur in PPG signals recorded from patients with OSA [39]. Consequently, it is important that algorithms used to analyse PPG signals during PSG remain robust during irregular heart rhythms.

This study aimed to develop a PPG signal-quality assessment algorithm for robust PAT estimation, and investigate the influence of signal quality on PAT estimation during various sleep stages and events. Since the ECG is recorded synchronously with the PPG during PSG, the proposed algorithm uses information from the ECG signal for PPG signal-quality assessment. Specifically, the proposed algorithm determines approximate locations of PPG pulse waves onsets from the locations of R and T waves in the ECG.

The investigation of this study consists of the following stages: (i) PPG signal-quality assessment in different sleep stages and events related to sleep-disordered breathing; (ii) PAT estimation in high- and low-quality PPG segments and during regular and irregular rhythms; (iii) PAT estimation in different sleep stages and events related to sleep-disordered breathing.

## 2. Materials and Methods

### 2.1. Data

**Figure 1:**
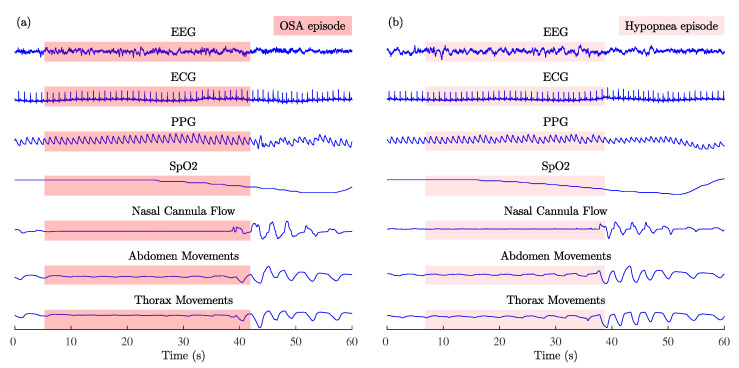
Examples of MESA multichannel physiological signals recorded during (**a**) OSA and (**b**) hypopnea episodes: electroencephalogram (EEG), electrocardiogram (ECG), photoplethysmogram (PPG), arterial blood oxygen saturation (SpO2), nasal cannula flow, abdomen, and thorax movements.

In this study, the Multi-Ethnic Study of Atherosclerosis (MESA) dataset [40,41] was used to implement and investigate the PPG signal-quality assessment algorithm. The MESA dataset contains data from 2055 patients aged 54–95 years old, totaling 16,300 h full overnight annotated PSG signals. Recordings were performed at home using the Compumedics Somte system. ECG and PPG signals with a sampling rate of 256 Hz were analyzed. PPG signals were recorded from the finger using the Nonin 8000 sensor. Figure 1 shows examples of MESA multichannel physiological signals recorded during OSA and hypopnea episodes.

The quality of PPG signals was analyzed in following annotated categories of the MESA dataset:

(1) sleep stages—NREM1, NREM2, NREM3, REM, and the wake stage;

(2) sleep-disordered breathing related events—OSA, hypopnea, SpO2 desaturations, and arousal episodes.

### 2.2. ECG & PPG Pre-Processing

The ECG signal was filtered by a zero-phase forth-order Butterworth low-pass filter with a cut-off frequency of 25 Hz to remove high-frequency interference. The baseline was removed to facilitate detection of ECG waves as follows [42]. The baseline was calculated by using a median filter with overlapping windows of 1 s duration and 0.5 s overlap. This was interpolated and subtracted from the ECG signal.

The PPG signal was filtered by a zero-phase forth-order Butterworth band-pass filter with a pass-band of 0.4–6 Hz.

### 2.3. PPG Signal-Quality Assessment

**Figure 2:**
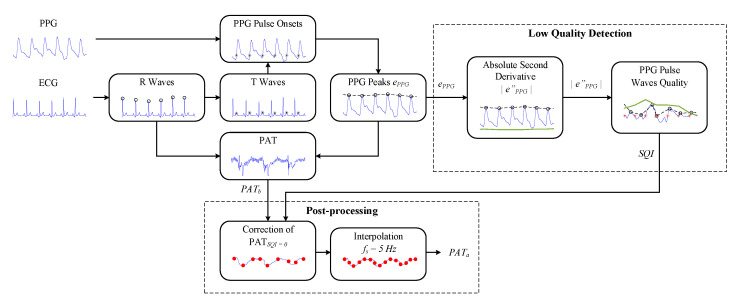
The block diagram of the PPG signal-quality assessment algorithm for robust PAT estimation.

The implemented PPG signal-quality assessment algorithm for robust PAT estimation (see Figure 2) has five stages: (i) identifying R and T waves in ECG signals; (ii) determining the locations of PPG pulse onsets; (iii) assessing the variability in PPG pulse amplitudes; (iv) identifying low-quality PPG pulse waves; and (v) robust PAT estimation. A pseudocode of the implemented ECG-guided PPG signal-quality assessment algorithm is presented in Algorithm 1.
**Algorithm 1** ECG-Guided PPG Signal-Quality Assessment.  1:**procedure** Identifying R and T Waves in ECG Signals  2:    ECG→R waves→R waves positions  3:    R waves positions→T waves positions  4:**procedure** Determining the Locations of PPG Pulse Onsets  5:    PPG onsets = min(PPG(R waves positions:T waves positions))  6:    PPG onsets→PPG onsets positions  7:**procedure** Assessing the Variability in PPG Pulse Amplitudes  8:    ***step 1:** PPG Envelope Estimation*  9:    **for** *i = 1: length(PPG onsets positions) - 1* **do**10:        $e_{PPG}\left(i\right)$ = max(PPG(PPG onsets positions(*i*): PPG onsets positions(*i*+1)))11:    **end**12:    ***step 2:** Absolute Second Derivative of PPG Envelope Estimation*13:    **for** *i = 1: length($e_{PPG}$) - 2* **do**14:        $|{e^{\prime \prime }}_{PPG}\left(i\right)|\;=\;|e_{PPG}\left(i+2\right)-2\cdot e_{PPG}\left(i+1\right)+e_{PPG}\left(i\right)|$15:    **end**16:    ***step 3:** Absolute Second Derivative of PPG Envelope Smoothing*17:    order=518:    $|{e^{\prime \prime }}_{PPG}{\left(i\right)|}_{s}$ = *median filter($|{e^{\prime \prime }}_{PPG}\left(i\right)|$, order)*19:    ***step 4:** Absolute Second Derivative of PPG Envelope Offsetting*20:    *k-subtraction constant*; $N\;=\;length(|{e^{\prime \prime }}_{PPG}{|}_{s})$21:    $|{e^{\prime \prime }}_{PPG}{|}_{center}\;=\;{\left|{e^{\prime \prime }}_{PPG}\right|}_{s}\left(round\left(0.25\cdot N\right):round\left(0.75\cdot N\right)\right)$22:    $|{e^{\prime \prime }}_{PPG}{\left(i\right)|}_{o}\;=\;|{e^{\prime \prime }}_{PPG}{\left(i\right)|}_{s}-k\cdot mean(|{e^{\prime \prime }}_{PPG}{|}_{center})$23:**procedure** Identifying Low-Quality PPG Pulse Waves24:    *thresholds:* $\theta_{1}$ and $\theta_{2}$25:    **if** $\left(\right|{e^{\prime \prime }}_{PPG}{|}_{o}\;<\;\theta_{1})\;\&\left(e_{PPG}\;>\;\theta_{2}\right)$ **then**26:    SQI=1-High Quality27:    **otherwise**28:    SQI=0-Low Quality29:    **end**

#### 2.3.1. Identifying R and T Waves in ECG Signals

The R-DECO algorithm [43,44,45] was used to detect R waves in ECG signals. Due to its small number of operations and simplicity, the algorithm is particularly suitable for processing long-term recordings, such as ECG signals registered during PSG. The R-DECO algorithm has been found to perform well, with sensitivity of 99.6% and positive predictive value of 99.7% [43] in the MIT/BIH arrhythmia database [46].

**Figure 3:**
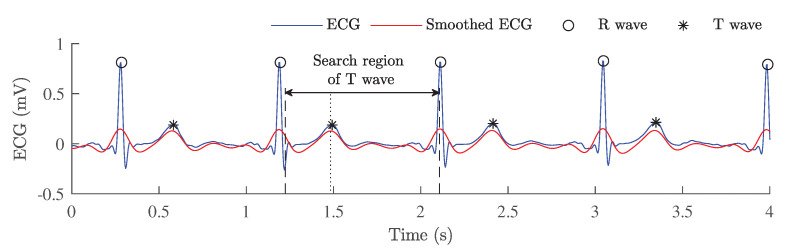
A low-complexity detection of T waves from the ECG signal by using additional filtering.

After detection of R waves, a low-complexity detection of T waves was performed (see Figure 3). It was observed that this implemented approach works more accurately and efficiently on MESA ECG signals than other open-access algorithms to detect T waves [47,48]. To facilitate detection of the T wave, the smoothed ECG signal was obtained by additionally filtering the prepossessed ECG. This was performed using a zero-phase 4th order Butterworth band-pass filter with a pass-band of 1–6 Hz. In the next step, the position of the T peak was located in a search region of the smoothed ECG signal. It was assumed that the approximate duration of the RS interval is equal to half of the maximum duration of the QRS complex in the normal range, i.e., 0.12 s [49]. Therefore, to detect T waves as accurately as possible, a search region was limited from the approximate position of the S peak till the following R wave and defined as [$R_{i}$ + 0.06(s): $R_{i+1}$]. This limitation in the smoothed ECG helped us to avoid misattributions of T waves. Then the position of the first peak in a search region was identified as the position of the T wave.

#### 2.3.2. Determining the Locations of PPG Pulse Onsets

The locations of PPG pulse onsets were determined as PPG minimum points between ECG R and T waves. The pulse onset is caused by the heart starting to pump blood into the vessels, whereas the ECG T wave reflects ventricular repolarization, and its location is nearby with that of the PPG pulse onset [50,51].

#### 2.3.3. Assessing the Variability in PPG Pulse Amplitudes

It might be complicated to exploit pulse intervals for assessing the quality of the PPG signal characterized by an irregular pattern of cardiac activity. For instance, pulse intervals vary greatly during arrhythmia, thus their use could result in PPG signals collected during arrhythmia being falsely classified as low quality. The alternative proposed approach is to use the finite differences of the PPG signal envelope.

First, the absolute second derivative of the PPG signal envelope, $|{e^{\prime \prime }}_{PPG}|$, was calculated as follows. The PPG signal envelope, $e_{PPG}$, was defined as the maximum value of the PPG signal in the delineated interval between each pair of adjacent pulse onsets (see Algorithm 1 description for further details). Then, $e_{PPG}$ was absolutely differentiated twice (i.e., the absolute second derivative $|{e^{\prime \prime }}_{PPG}|$ of the PPG signal envelope, $e_{PPG}$) as described below:

(1) $$|{e^{\prime \prime }}_{PPG}\left(i\right)|\;=\;|e_{PPG}\left(i+2\right)-2\cdot e_{PPG}\left(i+1\right)+e_{PPG}\left(i\right)|,$$

where $e_{PPG}$ is the PPG signal envelope, $|{e^{\prime \prime }}_{PPG}|$ is the absolute second derivative of the $e_{PPG}$, and *i* is a beat index.

Second, $|{e^{\prime \prime }}_{PPG}|$ was processed for analysis. To do so, $|{e^{\prime \prime }}_{PPG}|$ was smoothed by using a fifth-order median filter, and then $|{e^{\prime \prime }}_{PPG}{|}_{s}$ was obtained. In addition, $|{e^{\prime \prime }}_{PPG}{|}_{s}$ was offset as follows. In most cases, the initial and final segments of PSG signals, especially signals sensitive to motion artifacts such as PPG, were corrupted as subjects were often awake at these times. Since the analysis of the PPG signal was off-line, in order to separate the DC component of $|{e^{\prime \prime }}_{PPG}{|}_{s}$, the DC component was calculated as the mean value of a central 50% of the signal. Only a central part of the signal was selected due to observed PPG quality issues in the initial and final segments of PSG signals, thus allowing a more robust assessment of the mean value of $|{e^{\prime \prime }}_{PPG}{|}_{s}$. $|{e^{\prime \prime }}_{PPG}{|}_{s}$ was then offset by subtracting the estimated DC component from it *k* times, and then $|{e^{\prime \prime }}_{PPG}{|}_{o}$ was calculated. The procedure is described as follows:

(2) $$|{e^{\prime \prime }}_{PPG}{\left(i\right)|}_{o}\;=\;{\left|{e^{\prime \prime }}_{PPG}\left(i\right)\right|}_{s}-2\cdot k\cdot \frac{\sum_{i\;\approx \;0.25\cdot N}^{\;\approx \;0.75\cdot N}|{e^{\prime \prime }}_{PPG}{\left(i\right)|}_{s}}{N},$$

where *k* is the subtraction constant for offsetting, *N* is the length of the $|{e^{\prime \prime }}_{PPG}{|}_{s}$, and *i* is a beat index.

#### 2.3.4. Identifying Low-Quality PPG Pulse Waves

Two thresholds, $\theta_{1}$ and $\theta_{2}$, were used to identify low-quality pulses in PPG. The first threshold $\theta_{1}$ was defined as the limit value of the $|{e^{\prime \prime }}_{PPG}{|}_{o}$. The second threshold $\theta_{2}$ was defined as the limit value of the PPG signal envelope, $e_{PPG}$. The segmentation of low-quality pulse waves is described as follows:

(3) $$SQI\;=\;\left\{\begin{array}{cc} 1, & \mathrm{if}\;(|{e^{\prime \prime }}_{PPG}{|}_{o}\;<\;\theta_{1})\;\&\left(e_{PPG}\;>\;\theta_{2}\right)\\ 0, & \mathrm{otherwise},\; \end{array}\right.$$

where signal quality index SQI = 1 indicates that a PPG pulse wave was of high quality, and SQI = 0 indicates low quality.

**Figure 4:**
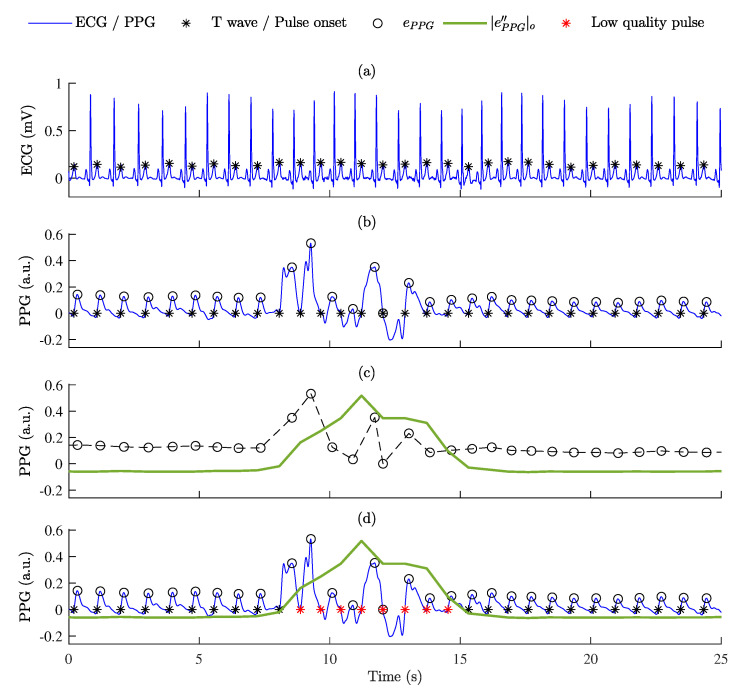
The segmentation of low-quality PPG pulse waves: (**a**) ECG signal with detected T waves; (**b**) PPG signal with detected pulse onsets and estimated maximum values of the PPG signal envelope, $e_{PPG}$ (black circles); (**c**) the second derivative of the PPG signal envelope after offsetting, $|{e^{\prime \prime }}_{PPG}{|}_{o}$ (shown in green) was calculated from the PPG signal envelope, $e_{PPG}$ (black circles); (**d**) the identification of low-quality pulses according to thresholds $\theta_{1}$ and $\theta_{2}$ (red stars indicate low- quality pulses).

Figure 4 shows an example of the segmentation of low-quality PPG pulse waves.

### 2.4. PAT Estimation

**Figure 5:**
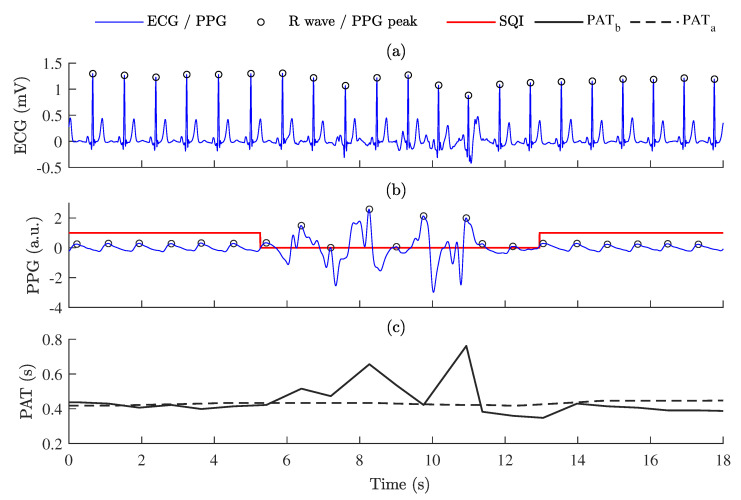
The relationship between PPG signal quality and PAT: (**a**) ECG signal; (**b**) PPG signal with labeled signal quality (SQI); (**c**) PAT variations estimated as time intervals between ECG R peaks and PPG systolic peaks—PAT before post-processing, $PAT_{b}$, and PAT after post-processing, $PAT_{a}$.

PAT was estimated as the time interval between the ECG R peak and the PPG systolic peak. It was decided to use the PPG systolic peak for PAT estimation, rather than the PPG pulse onset because it allowed us to obtain more noticeable dipping pattern of PAT during apnea events. The PPG systolic peak was defined as the maximum value of the PPG signal in the delineated interval between each pair of adjacent pulse onsets, which were determined as described in Section 2.3.2. Figure 5 shows an example of PAT variations and its relations with estimated PPG signal quality.

### 2.5. PAT Post-Processing

PAT post-processing consisted of correcting low-quality intervals and interpolation of PAT sequences (see Figure 2). PAT values corresponding to low-quality PPG pulse waves were obtained as the mean of the *M* following PAT values as described below:

(4) $$PAT_{i}=\left\{\begin{array}{cc} \frac{\sum_{j=i+1}^{i+M}PAT_{j}}{M}, & \mathrm{if}\;SQI\;=\;0\\ PAT_{i}, & \mathrm{otherwise},\; \end{array}\right.$$

where *i* is a beat index, and *M* is the order of averaging.

Additionally, obtained PAT sequences were processed by using a *n*-order median filter.

As PAT was estimated for each pulse-to-pulse interval and indexed by *i*, where *i* is the order of the beat occurring at time *t*(*i*), modified Akima cubic Hermite interpolation method [52] was used to obtain a PAT signal uniformly sampled in time with a sampling rate of 5 Hz. Figure 5c shows PAT before post-processing, $PAT_{b}$, and after post-processing, $PAT_{a}$, respectively.

### 2.6. PAT Changes during Sleep-Disordered Breathing

**Figure 6:**
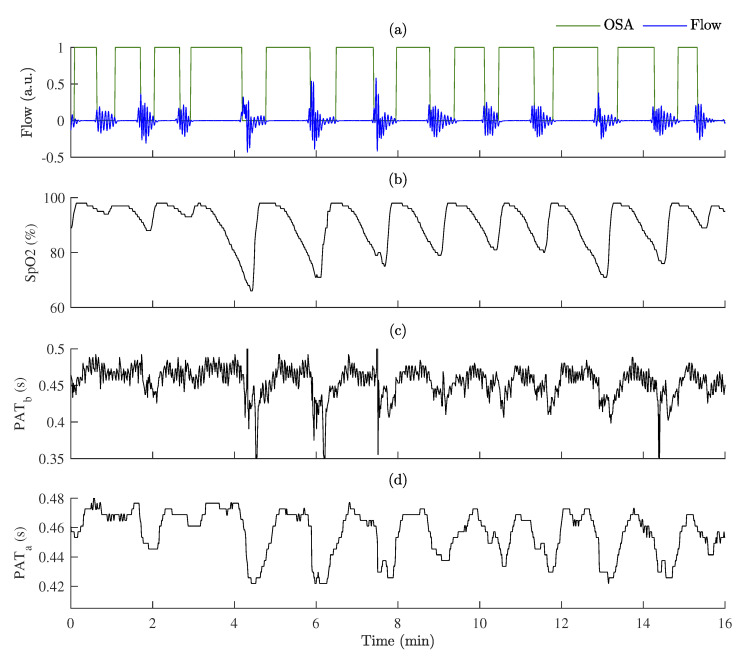
An example of PAT variations during OSA episodes: (**a**) respiration flow signal with labeled OSA episodes; (**b**) SpO2 variations; (**c**) PAT before post-processing, $PAT_{b}$; (**d**) PAT after post-processing, $PAT_{a}$.

PAT changes during OSA episodes are illustrated in Figure 6. Additionally, variations in SpO2 are provided for comparison. Based on SpO2 variability, we can see that after PPG quality-based post-processing the dipping pattern of PAT is distinguished more obviously. More precisely estimated dipping pattern shows that PAT tended to decrease significantly more after the OSA episode or at the end of the event than during it.

After post-processing obtained PAT estimates were compared with SpO2. The percentage change was calculated as the range of values, divided by the maximum value. The changes during and 30 s after OSA and hypopnea episodes were assessed for PAT and SpO2.

### 2.7. Irregular Rhythm Detection

Intervals of irregular rhythm were identified in order to investigate the signal quality and PAT distributions during regular and irregular beat-to-beat intervals. To do so, a fast and simple low-complexity algorithm for continuous long-term monitoring [53] was used to detect irregular rhythms, based on sequences of RR intervals from ECG signals. This detector has been found to perform well on the MIT–BIH database (sensitivity of 97.1%, specificity of 98.3%) [53]. The detector consists of ectopic beat filtering, bigeminal suppression, characterization of RR interval irregularity, and signal fusion [53]. Thus, it is based on the observation that atrial fibrillation episodes have increased RR irregularity and usually are associated with increased heart rate. The algorithm was implemented with just a few arithmetic operations per beat, using an 8-beat sliding window [53].

The last annotated segment of the wake stage at the end of PSG tests, during which a subject was already fully awake, was not analyzed for irregular rhythm detection.

### 2.8. Comparison of PPG Signal-Quality Assessment Algorithms

Our ECG-guided PPG signal-quality assessment algorithm was compared with the template matching (TM) approach proposed by Orphanidou et al. [28]. The TM-based approach searches for regularity in a segment which is an indicator of reliability, since a segment contaminated by artefact would be irregular in morphology [28]. This algorithm consists of [28]: (i) estimation of median beat-to-beat interval by using all the detected PPG pulse peaks of each sample; (ii) extraction of individual PPG pulse waves by using a window, the width of which is the median beat-to-beat interval, centered on each detected PPG pulse peak; (iii) generation of the mean PPG pulse wave template by taking the mean of all PPG pulse waves in the sample; (iv) estimation of the correlation coefficient of each individual PPG pulse wave with the obtained average template; and (v) estimation of the average correlation coefficient obtained by averaging all correlation coefficients over the whole PPG sample. In this work, the TM-based algorithm uses a 10 s window in order to assess the quality of the PPG.

### 2.9. Statistical Analysis

The quality of PPG signals was assessed by using two algorithms on the nine categories of MESA data mentioned in Section 2.1: NREM1, NREM2, NREM3, REM, and wake stages; OSA, hypopnea, SpO2 desaturation, and arousal episodes.

Additionally, the computational efficiency of both algorithms was assessed by measuring the time taken to analyze the PPG signals (with approximate duration of 12 h). The main parameters of the computer used for this purpose are provided as follows: processor—AMD Ryzen Threadripper PRO 5995WX 64-Cores, 2.70 GHz, 64-bit operating system, RAM—128 GB.

The Anderson–Darling test found no Gaussian distribution in the analyzed data. The non-parametric paired Wilcoxon signed rank test was used to test for statistical differences between: (i) proportions of high-quality PPG in different sleep stages and events related to sleep-disordered breathing; (ii) interquartile ranges of PAT distributions in high- and low-quality PPG segments/regular and irregular rhythms, and proportions of high-quality PPG in them; (iii) interquartile ranges of PAT distributions in different sleep stages and events related to sleep-disordered breathing; (iv) percentage changes of PAT and SpO2 during and after apnea event. Additionally, the effect size was estimated by using matched-pairs rank biserial correlation coefficient $r_{c}$ values [54]. For this purpose, median values for each subject were obtained and compared. The effect size is considered small when matched-pairs $r_{c}$ < 0.30, medium—$r_{c}$ ≥ 0.30, and large—$r_{c}$ ≥ 0.50 [55].

## 3. Results

### 3.1. Parameter Settings

In order to detect low-quality pulses in PPG, after $|{e^{\prime \prime }}_{PPG}{|}_{s}$ offsetting, the first threshold was defined as $\theta_{1}$ = 0, assuming that no amplitude disturbances in high-quality PPG exists. The second threshold was defined with assumption as $\theta_{2}$ = 0.0005, the limit value of the PPG signal envelope, $e_{PPG}$, with which it is still possible to reliably investigate the morphology of the PPG pulse waveform. The subtraction constant for $|{e^{\prime \prime }}_{PPG}{|}_{s}$ offsetting (see Equation (2)) was selected *k* = 3, allowing $\theta_{1}$ = 0 to be meaningful for distinguishing low-quality PPG segments correctly in most cases.

The definition of the subtraction constant, *k*, is related to the threshold, $\theta_{1}$. The aim was to subtract the mean value of the $|{e^{\prime \prime }}_{PPG}{|}_{s}$ so many times that after this kind of offsetting the values of the obtained $|{e^{\prime \prime }}_{PPG}{|}_{o}$ in high-quality PPG intervals would be less than the first threshold, $\theta_{1}$. By using grid search within the range *k* = 0.5 ÷ 5, the first such determined value satisfying these conditions was *k* = 3.

The second threshold, $\theta_{2}$, was used to exclude low-quality PPG segments with distorted morphology due to possible poor contact of the sensor with the skin. By investigating four cases—$\theta_{2}$ = 0.05, 0.005, 0.0005, 0.00005, we found that $\theta_{2}$ = 0.0005 allows us to distinguish these distorted PPG segments from high-quality PPG intervals correctly.

The orders of averaging and median filtering for PAT sequences were selected *M* = 5 and *n* = 15, respectively, which allowed us to significantly reduce deviations in PAT beat-to-beat intervals of long-term signals.

The linear and non-linear averaging in PAT post-processing reduces outliers occurring due to low PPG signal-quality pulses. The order of linear averaging, *M*, was selected with the assumption that the PAT value corresponding to a low-quality PPG pulse should be approximately similar to the PAT values of the adjacent five pulses with a high quality. The order of non-linear filter was also selected empirically to *n* = 15 as it is a maximum value still preserving the PAT dipping pattern correlated with desaturations during apnea events without losing specific characteristics. We opted to use both linear and non-linear filters for post-processing of PAT time series. Since the linear averager is suitable for removing high frequency variations, while the median averager is suitable for removing outliers with the preservation of steep slopes of PAT signals.

### 3.2. Signal Quality of PPG in Different Sleep Stages and Sleep-Disordered Breathing Events

The proportion of sleep time in the MESA data was 42.6 ± 13.5% awake and 57.3 ± 13.5% asleep. The average number of events related to sleep-disordered breathing across all subjects was: 22.7 ± 45.8 OSA, 192.4 ± 118.9 hypopnea, 331.3 ± 171.6 oxygen desaturation, and 125.3 ± 96.3 arousal.

**Figure 7:**
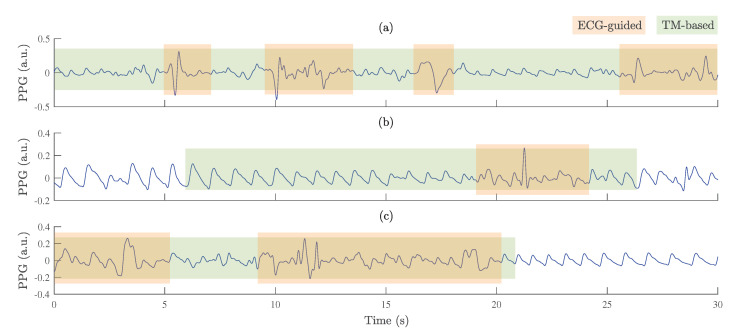
Examples of PPG signals with labeled poor-quality segments obtained by ECG-guided and TM-based algorithms: (**a**) wake, (**b**) oxygen desaturation, and (**c**) arousal.

The proportions of high-quality PPG pulse waves across all subjects in each category were obtained by using the ECG-guided algorithm: 80.9% whilst awake, 98.8% whilst asleep (NREM1, NREM2, NREM3, and REM), 99.4% during apnea (OSA and hypopnea), 93.3% during oxygen desaturation, and 88.9% during arousal. This indicates that PPG signals were mostly of higher quality during sleep and apnea episodes, but of lower quality whilst awake and during oxygen desaturation and arousal events associated with sleep-disordered breathing. Figure 7 shows examples of PPG signals in wake, oxygen desaturation, and arousal segments, in which the proportions of high-quality PPG were the lowest. Additionally, low-quality segments of PPG signals classified by ECG-guided and TM-based algorithms were compared with each other. It might be observed that the ECG-guided algorithm tends to detect large amplitude variations quite well and classify them as poor quality, but in some cases it classifies segments with low amplitude disturbances as high quality. Meanwhile, the TM-based algorithm often classifies PPG pulse waves of high-quality as poor-quality intervals.

**Figure 8:**
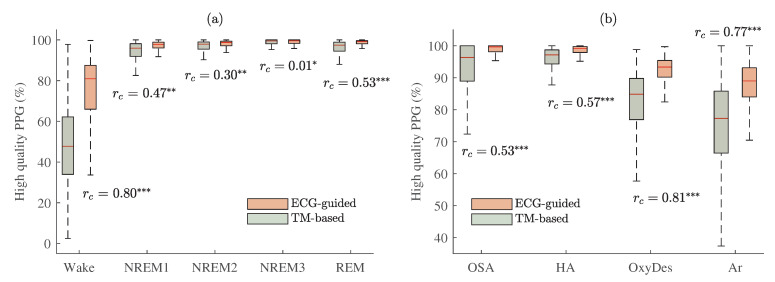
The proportion of PPG pulse waves classified as high quality by the two signal-quality assessment algorithms with estimated matched-pairs $r_{c}$ values: ECG-guided and TM-based approaches. Results are shown (**a**): whilst awake and for different sleep stages; (**b**) for different events related to sleep-disordered breathing. *p* < 0.001 and $r_{c}$ < 0.30 is marked ${}^{*}$, *p* < 0.001 and $r_{c}$≥ 0.30—${}^{\;**}$, *p* < 0.001 and $r_{c}$≥ 0.50—${}^{\;***}$.

The proportions of PPG pulse waves classified as high quality by the ECG-guided and the reference TM-based algorithms are shown for different sleep stages in Figure 8a, and for different events in Figure 8b. For six out of the nine categories a large effect size was obtained (*p* < 0.001). This means that the proportion of high-quality PPG in these segments obtained by using the TM-based approach is much lower comparing it with the ECG-guided algorithm.

The matched-pairs $r_{c}$ values of proportions of high-quality PPG between different sleep stages and events related to sleep-disordered breathing are provided in Table 1 and Table 2, respectively (*p* < 0.001 and $r_{c}$ < 0.30 is marked ${}^{*}$, *p* < 0.001 and $r_{c}$ ≥ 0.30—${}^{\;**}$, *p* < 0.001 and $r_{c}$ ≥ 0.50—${}^{\;***}$). Table 1 and Figure 8a show that the proportion of high-quality PPG was significantly lower in the wake stage than in different sleep stages (*p* < 0.001, $r_{c}$ > 0.90). According to Table 2 and Figure 8b, it can be seen that the proportions of high-quality PPG were significantly lower in apnea-related oxygen desaturation (*e.g*, with the ECG-guided algorithm obtained $r_{c}$ = 0.88 and $r_{c}$ = 0.97, compared to OSA and hypopnea, respectively, when *p* < 0.001) and arousal ($r_{c}$ = 0.93 and $r_{c}$ = 0.98, when *p* < 0.001) than in apnea events. As well as, the effect sizes were larger in most cases when the quality of PPG signals was assessed by using the TM-based approach than the ECG-guided algorithm.

In terms of computational efficiency, the ECG-guided algorithm took an average of 11.6 s, whereas the TM-based algorithm result was 90.1 s for quality assessment through one signal. Therefore, the ECG-guided algorithm took approximately one eighth of the time of the TM-based approach.

### 3.3. PAT in High & Low-Quality PPG and Regular & Irregular Rhythms

**Figure 9:**
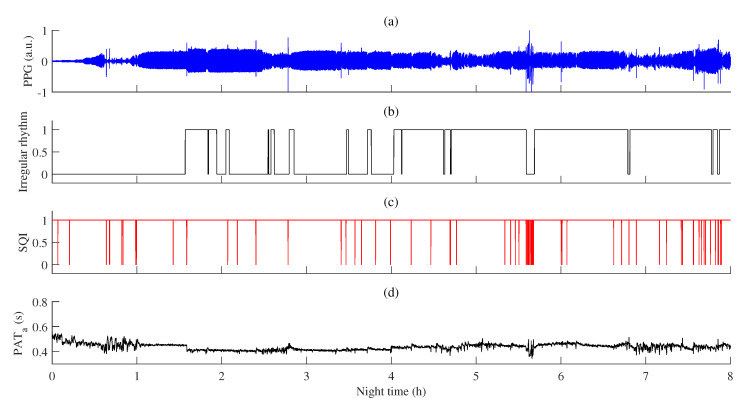
An example of PAT variations during regular and irregular rhythms: (**a**) PPG signal; (**b**) detected episodes of irregular rhythm (1—irregular rhythm, 0—regular rhythm); (**c**) PPG signal quality estimated by the ECG-guided algorithm, SQI (SQI = 1—high-quality, SQI = 0—low-quality); (**d**) PAT after post-processing, $PAT_{a}$.

An example of PAT variations during regular and irregular rhythms is provided in Figure 9. In addition, the PPG signal recorded during throughout PSG and signal quality estimated by the ECG-guided algorithm are shown.

Since we did not have reference PAT, the way to test the effect of the signal-quality assessment is to study the variations in PAT estimates, assuming that low-quality PPG pulse waveforms lead to outlier PAT values. Therefore, interquartile ranges of PAT were analyzed in order to investigate the relationship between PPG signal quality and deviations in PAT.

**Figure 10:**
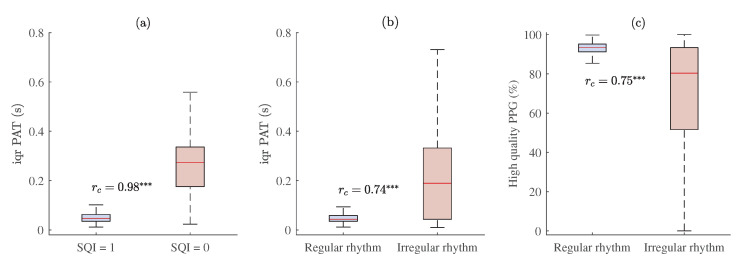
The relationship between PPG signal quality and PAT with estimated matched-pairs $r_{c}$ values. Interquartile range of PAT across all MESA subjects in: (**a**) high- (SQI = 1) and low-quality (SQI = 0) PPG segments, and (**b**) regular and irregular rhythm PPG beat-to-beat intervals; (**c**) the proportion of high-quality PPG during regular and irregular rhythm PPG pulse waves. *p* < 0.001 and $r_{c}$≥ 0.50 is marked ${}^{***}$.

Interquartile ranges of PAT distributions across all 2055 MESA subjects in high- (SQI = 1) and low-quality (SQI = 0) PPG segments are provided in Figure 10a, in which it can be seen that PPG signal quality caused deviations in PAT. For instance, interquartile range of PAT was statistically higher (*p* < 0.001, $r_{c}$ = 0.98) in low- rather than high-quality PPG segments.

The proportion of high-quality PPG during irregular rhythm was statistically lower (*p* < 0.001, $r_{c}$ = 0.75) than during regular rhythm intervals (see Figure 10c), which also might be related with a statistically higher (*p* < 0.001, $r_{c}$ = 0.74) interquartile range of PAT during irregular rhythm pulse waves (see Figure 9 and Figure 10b).

### 3.4. PAT in Different Sleep Stages & Sleep-Disordered Breathing Events

**Figure 11:**
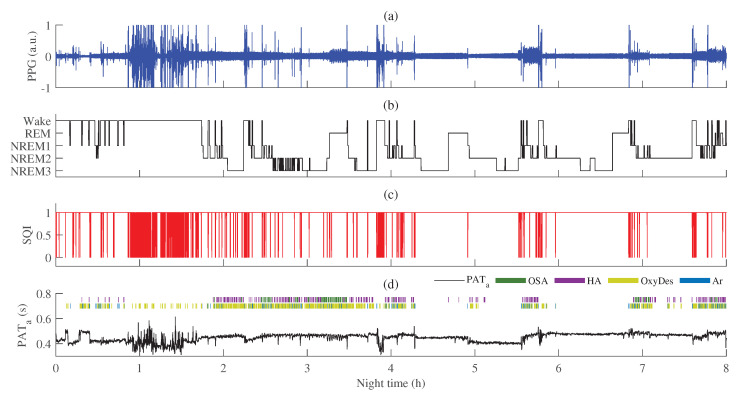
An example of PAT variations in different sleep stages: (**a**) PPG signal; (**b**) recorded hypnogram during PSG; (**c**) PPG signal quality estimated by the ECG-guided algorithm, SQI (SQI = 1—high-quality, SQI = 0—low-quality); (**d**) PAT after post-processing, $PAT_{a}$, with annotated obstructive sleep apnea (OSA), hypopnea (HA), oxygen desaturation (OxyDes), and arousal (Ar) episodes.

An example of PAT variations in different sleep stages (wake, NREM1, NREM2, NREM3, REM) and during sleep-disordered breathing events (OSA, hypopnea, oxygen desaturation, and arousal) is provided in Figure 11. Additionally, the PPG signal and signal quality estimated by the ECG-guided algorithm are shown.

**Figure 12:**
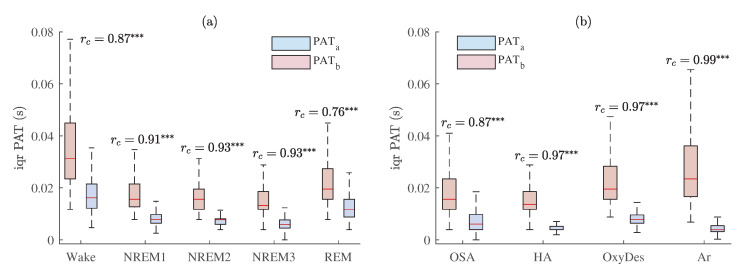
The interquartile range of PAT across all MESA subjects with estimated matched-pairs $r_{c}$ values in: (**a**) different sleep stages; (**b**) different events related to sleep-disordered breathing. Results are shown before and after PAT post-processing, $PAT_{b}$ and $PAT_{a}$, respectively. *p* < 0.001 and $r_{c}$≥ 0.50 is marked ${}^{***}$.

Interquartile ranges of PAT distributions in different sleep stages (wake, NREM1, NREM2, NREM3, REM) and in different events related to sleep-disordered breathing (OSA (obstructive sleep apnea), HA (hypopnea), OxyDes (SpO2 desaturation), and Ar (arousal episodes)) are provided in Figure 12a and b, respectively.

Comparing differences between distributions of different sleep stages (see Figure 12a) and events related to sleep-disordered breathing (see Figure 12b), interquartile ranges of PAT were the largest during wake (see Figure 11), oxygen desaturation, and arousal events, in which the proportions of high-quality PPG were the lowest. However, after post-processing and eliminating PAT measurements derived from low-quality PPG signals deviations in PAT decreased, especially, in hypopnea, oxygen desaturation, and arousal segments ($r_{c}$ = 0.97, $r_{c}$ = 0.97, $r_{c}$ = 0.99, respectively, when *p* < 0.001). The decrease in deviations of PAT could be explained by the fact that a lower quality of the PPG signal results in a higher variability of PAT estimates.

The matched-pairs $r_{c}$ values of interquartile ranges of PAT distributions between different sleep stages and events related to sleep-disordered breathing are provided in Table 3 and Table 4, respectively (*p* < 0.001 and $r_{c}$ < 0.30 is marked ${}^{*}$, *p* < 0.001 and $r_{c}$ ≥ 0.30—${}^{**}$, *p* < 0.001 and $r_{c}$ ≥ 0.50—${}^{***}$). According to Table 3 and Table 4, it can be seen that PAT post-processing allows to obtain larger differences between different distributions in most cases. This is especially true during sleep stages, where PAT assessment might be used as a supplementary tool for hypnogram evaluation.

**Figure 13:**
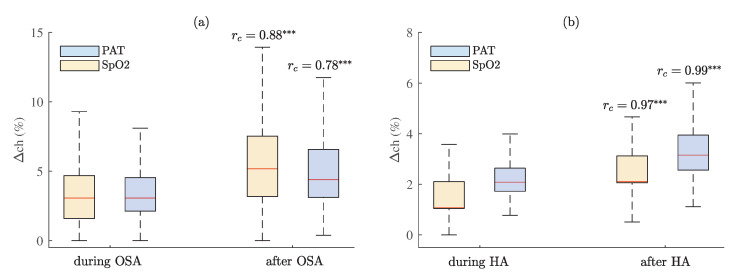
The percentage changes of PAT and SpO2 during and 30 s after (**a**) OSA and (**b**) hypopnea episodes across all subjects with estimated matched-pairs $r_{c}$ values. *p* < 0.001 and $r_{c}$≥ 0.50 is marked ${}^{\;***}$.

The percentage changes of PAT during and 30 s after OSA and hypopnea episodes across all subjects are provided in Figure 13. Additionally, the percentage changes of SpO2 are provided for comparison. The results show that the percentage change is significantly higher after apnea rather than during apnea events. Statistically higher differences were obtained when comparing the distributions related to hypopnea than OSA ($r_{c}$—0.97 > 0.88; 0.99 > 0.78, in SpO2 and PAT, respectively, when *p* < 0.001). However, the percentage changes of parameters were larger in value during and after OSA than hypopnea. For instance, median percentage changes of PAT were 3.1% > 2.1%, during OSA and hypopnea, respectively, and 4.4% > 3.2% after OSA and hypopnea, respectively. While median percentage changes of SpO2 were 3.1% > 1.1%, during OSA and hypopnea, respectively, and 5.2% > 2.1% after OSA and hypopnea, respectively.

## 4. Discussion

This study aimed to develop a PPG signal-quality assessment algorithm for robust PAT estimation, and investigate the influence of signal quality on PAT estimation during various sleep stages and events. The algorithm consisted of: (i) identifying R and T waves in ECG signals; (ii) determining the locations of PPG pulse onsets; (iii) assessing the variability in PPG pulse amplitudes; (iv) identifying low-quality PPG pulse waves; and (v) robust PAT estimation. The key findings were as follows. First, PPG signals were of mostly high quality during sleep and apnea episodes, but of lower quality whilst awake and during oxygen desaturation and arousal events. Second, a greater proportion of PPG signals were deemed to be of high quality when using the new approach compared to the reference algorithm. Third, a lower quality of the PPG signal was found to be significantly associated with a higher interquartile range of PAT distribution, which shows that assessing signal quality would lead to a more accurate PAT estimation. Fourth, PPG signal quality-based PAT post-processing reduced PAT deviations during PSG studies. Fifth, PAT tended to change significantly more after the apnea episode than during it, and these changes often correlate with SpO2.

Another study [56] also reveals that the highest quality of the PPG signal is observed during sleep (93.1% of the time). This is consistent with the results of our study, which showed that the majority of PPG signals during sleep were suitable for analysis. In addition, a lower PPG signal quality was observed during sleep-disordered breathing events, such as apnea-related oxygen desaturations and arousals, and during irregular rhythm episodes potentially related to arrhythmia.

The interquartile range of PAT distribution across all subjects was the highest in those segments in which the proportions of high-quality PPG were the lowest (see Figure 10a), i.e., wake, oxygen desaturation, and arousal events (see Figure 12). Furthermore, interquartile range of PAT during irregular rhythm was also higher than during regular rhythm beat-to-beat intervals (see Figure 10b), which corresponds to a lower proportion of high-quality signal during irregular rhythm PPG pulse waves (see Figure 10c).

The results (see Figure 12) also revealed that PPG signal quality-based PAT post-processing reduced PAT deviations (see Table 3 and Table 4). Thus, this study showed that assessing PPG signal quality can lead to a more accurate PAT estimation. Consequently, it is important to exclude low-quality PPG segments from PSG analyses before evaluating nocturnal blood pressure response from PAT. Since the ECG signal is less susceptible to artifacts, it makes sense to use the ECG for evaluation of PPG signal quality during PSG. Therefore, such PPG signal quality-based PAT evaluation could enable to reduce obtained deviations and be used to identify outliers of PSG-derived PAT. On the other hand, the information on the higher interquartile range of PAT might be used to detect episodes of insomnia and micro-arousals, or be a supplementary tool for hypnogram evaluation during PSG.

In terms of PAT variability during apnea events, the percentage change analysis (see Figure 13) showed that PAT tended to change significantly more after the apnea episode with some time delay than during it. Considering that the quality of PPG signals can be affected by arousals occurring after apnea episodes, quality analysis for PAT monitoring becomes crucial. It is worth mentioning that PPG quality-based post-processing helped to highlight the dipping pattern of PAT, which could provide a clinically useful aid for sleep apnea monitoring during PSG studies.

The implemented ECG-guided algorithm was compared with the reference TM-based approach [28] for PPG signal-quality assessment. In the obtained results (see Figure 8), we might see a correlation between the distributions of different stages, but in some cases (e.g., wake and arousal segments) the proportion of high-quality PPG obtained by using the TM-based approach is much lower comparing it with the distributions of the ECG-guided algorithm. This could be explained by the fact that the TM-based approach assesses the quality of the PPG by using a 10-s window. Thus, even if only a few seconds are low quality, all 10 s will be classified as low quality. Therefore, it might underestimate the amount of high-quality data. Furthermore, it is important to note that the ECG-guided algorithm can be tuned to be more or less restrictive by changing the subtraction constant *k* or thresholds values, $\theta_{1}$ and $\theta_{2}$, respectively.

It is important to mention that the TM-based approach is based on the assumption that in a high-quality PPG signal, all the pulse waves are similar in shape. However, this is not always the case. For instance, during cardiac arrhythmia, the pulse waves differ in duration and shape, even in a high-quality recording. These differences are caused by the physiology rather than by poor quality signals. Therefore, perhaps the TM-based approach is erroneously classifying PPG signals during irregular heart rhythms as low quality, when they should be classified as high quality.

Regarding computational efficiency, the implemented ECG-guided algorithm requires less computations than the reference algorithm, which is one of the advantages of our proposed approach. Thus, it would be more suitable to analyze long-term PPG signals rather than the TM-based approach.

By assessing the quality of PPG signals during PSG, not only PAT could be precisely estimated. For instance, PPG signals could be used to evaluate pulse rate, its variability [57,58,59], and the morphological characteristics of pulse waves [60,61,62]. In this way, the PPG signal could provide information on myocardial function, the cardiovascular system and its components [2,57,58,59,63,64], blood perfusion [65], the balance of autonomic nervous system [60,61,66], and respiratory activity [67,68].

The proposed algorithm has several limitations. The first limitation is the dependence of the parameters *k*, $\theta_{1}$, $\theta_{2}$ of the algorithm on a specific equipment to record the PPG signal. Different amplifications and filtering of the PPG signal require new settings for the parameters *k*, $\theta_{1}$, and $\theta_{2}$. The algorithm could be modified to use normalized PPG signals in the future. Another limitation is the assumption that the T wave in the ECG is always present. However, in myocardial ischemia or injury, during electrolyte imbalances, or due to the use of certain medications, the T wave could be flat. In this case, the algorithm should be forced to switch to the mode of “No T wave detected” and relay only on PPG signal-based fiducial point detection.

## 5. Conclusions

To our knowledge, the quality of PPG signals during sleep stages and sleep-disordered breathing events has not previously been assessed. Our study revealed that PPG signals are of high quality during sleep and apnea events, whereas they are of lower quality whilst awake and during apnea-related oxygen desaturations and arousals. As well as, a lower quality of the PPG signal was found to be significantly associated with a higher interquartile range of PAT. Therefore, the implemented algorithm has a potential to increase the robustness of PAT estimation in PSG studies related to nocturnal blood pressure monitoring.

## Figures and Tables

**Table 1:** The matched-pairs $r_{c}$ values of proportions of high-quality PPG between different sleep stages obtained by using ECG-guided (orange background) and TM-based algorithms (green background). *p* < 0.001 and $r_{c}$ < 0.30 is marked ${}^{*}$, *p* < 0.001 and $r_{c}$≥ 0.30—${}^{**}$, *p* < 0.001 and $r_{c}$≥ 0.50—${}^{***}$.

Matched-Pairs $r_{c}$	Wake	NREM1	NREM2	NREM3	REM
Wake		$0.99^{\;***}$	$0.99^{\;***}$	$0.96^{\;***}$	$0.99^{\;***}$
NREM1	$0.99^{\;***}$		$0.49^{\;**}$	$0.58^{\;***}$	$0.60^{\;***}$
NREM2	$0.99^{\;***}$	$0.67^{\;***}$		$0.45^{\;**}$	$0.29^{\;*}$
NREM3	$0.99^{\;***}$	$0.83^{\;***}$	$0.72^{\;***}$		$0.19^{\;*}$
REM	$0.99^{\;***}$	$0.39^{\;**}$	$0.17^{\;*}$	$0.66^{\;***}$	

**Table 2:** The matched-pairs $r_{c}$ values of proportions of high-quality PPG between sleep-disordered breathing events obtained by using ECG-guided (orange background) and TM-based algorithms (green background), where OSA—obstructive sleep apnea, HA—hypopnea, OxyDes —oxygen desaturation, Ar—arousal events. *p* < 0.001 and $r_{c}$ < 0.30 is marked ${}^{*}$, *p* < 0.001 and $r_{c}$≥ 0.30—${}^{**}$, *p* < 0.001 and $r_{c}$≥ 0.50—${}^{***}$.

Matched-Pairs $r_{c}$	OSA	HA	OxyDes	Ar
OSA		$0.20^{\;*}$	$0.88^{\;***}$	$0.93^{\;***}$
HA	$0.32^{\;**}$		$0.97^{\;***}$	$0.98^{\;***}$
OxyDes	$0.72^{\;***}$	$0.99^{\;***}$		$0.62^{\;***}$
Ar	$0.87^{\;***}$	$0.97^{\;***}$	$0.61^{\;***}$	

**Table 3:** The matched-pairs $r_{c}$ values of interquartile ranges of PAT distributions between different sleep stages after (blue background) and before post-processing (red background). *p* < 0.001 and $r_{c}$≥ 0.30 is marked ${}^{**}$, *p* < 0.001 and $r_{c}$≥ 0.50—${}^{***}$.

Matched-Pairs $r_{c}$	Wake	NREM1	NREM2	NREM3	REM
Wake		$0.99^{\;***}$	$0.99^{\;***}$	$0.98^{\;***}$	$0.65^{\;***}$
NREM1	$0.97^{\;***}$		$0.38^{\;**}$	$0.69^{\;***}$	$0.87^{\;***}$
NREM2	$0.97^{\;***}$	$0.49^{\;**}$		$0.65^{\;***}$	$0.96^{\;***}$
NREM3	$0.94^{\;***}$	$0.54^{\;***}$	$0.42^{\;**}$		$0.95^{\;***}$
REM	$0.82^{\;***}$	$0.56^{\;***}$	$0.77^{\;***}$	$0.77^{\;***}$	

**Table 4:** The matched-pairs $r_{c}$ values of interquartile ranges of PAT distributions between different events related to sleep-disordered breathing after (blue background) and before post-processing (red background), where OSA—obstructive sleep apnea, HA—hypopnea, OxyDes—oxygen desaturation, Ar—arousal events. *p* < 0.001 and $r_{c}$ < 0.30 is marked ${}^{*}$, *p* < 0.001 and $r_{c}$≥ 0.30—${}^{**}$, *p* < 0.001 and $r_{c}$≥ 0.50—${}^{***}$.

Matched-Pairs $r_{c}$	OSA	HA	OxyDes	Ar
OSA		$0.69^{\;***}$	$0.34^{\;**}$	$0.70^{\;***}$
HA	$0.50^{\;***}$		$0.99^{\;***}$	$0.29^{\;*}$
OxyDes	$0.45^{\;**}$	$0.94^{\;***}$		$0.97^{\;***}$
Ar	$0.61^{\;***}$	$0.93^{\;***}$	$0.30^{\;**}$	

## Data Availability

Not applicable.

## Abbreviations

The following abbreviations are used in this manuscript:

Ar	Arousal episodes
EEG	Electroencephalogram
ECG	Electrocardiogram
EMG	Electromyogram
HA	Hypopnea
OSA	Obstructive sleep apnea
OxyDes	SpO2 desaturation
PAT	Pulse arrival time
PPG	Photoplethysmography
PSG	Polysomnography
SpO2	Arterial blood oxygen saturation
SQI	Signal-quality index
